# A cross-sectional study of the prevalence, barriers, and facilitators of cervical cancer screening in family planning clinics in Mombasa County, Kenya

**DOI:** 10.1186/s12913-022-08984-2

**Published:** 2022-12-23

**Authors:** McKenna C. Eastment, George Wanje, Barbra A. Richardson, Emily Mwaringa, Shem Patta, Kenneth Sherr, Ruanne V. Barnabas, Kishorchandra Mandaliya, Walter Jaoko, R. Scott McClelland

**Affiliations:** 1 Department of Medicine, University of Washington, Box 359909, 325 9th Avenue, Seattle, WA 98104 USA; 2 Department of Global Health, University of Washington, Seattle, WA USA; 3 Department of Biostatistics, University of Washington, Seattle, WA USA; 4 Vaccine and Infectious Disease Division, Fred Hutchinson Cancer Research Center, Seattle, WA USA; 5 Mombasa County Department of Health, Mombasa, Kenya; 6 Division of Infectious Diseases, Massachusetts General Hospital, Boston, MA USA; 7 Harvard Medical School, Boston, MA USA; 8 Department of Medical Microbiology and Immunology, University of Nairobi, Nairobi, Kenya; 9 Department of Epidemiology, University of Washington, Seattle, WA USA

**Keywords:** Cervical cancer screening, Integration, Family planning, Kenya

## Abstract

**Background:**

Cervical cancer is the most common cancer in sub-Saharan Africa. With appropriate screening and treatment, cervical cancer can be prevented. In Kenya, cervical cancer screening is recommended for all women of reproductive age who visit a health facility. In particular, the Kenyan Ministry of Health has tasked family planning clinics and HIV clinics with implementing cervical cancer screening as part of the overall cervical cancer screening strategy. A cross-sectional survey was conducted to understand cervical cancer screening practices and explore clinic-level barriers and facilitators to screening in family planning clinics (FP) in Mombasa County, Kenya.

**Methods:**

Structured interviews were conducted with randomly sampled FP clinic managers to collect information about clinic size, location, type, management support, infrastructure, screening practices, and availability of screening commodities. Data were abstracted from FP registers for a 15-month period from October 1, 2017 until December 31, 2018 to understand cervical cancer screening prevalence. Generalized linear models were used to calculate prevalence ratios (PR) and identify clinic-level correlates of reporting any cervical cancer screening.

**Results:**

A total of 70 clinics were sampled, 54% (38) were urban and 27% (19) were public facilities. The median number of staff in a clinic was 4 (interquartile range [IQR] 2–6) with a median of 1 provider trained to perform screening (IQR 0–3). Fifty-four percent (38/70) of clinic managers reported that their clinics performed cervical cancer screening. Of these, only 87% (33) and 71% (27) had dependable access to speculums and acetic acid, respectively. Being a public FP clinic was associated with higher prevalence of reported screening (14/38 [37%] vs 6/32 [16%]; prevalence rate ratio [PR] 1.57, 95%CI 1.05–2.33). Clinics that reported cervical cancer screening were much more likely to have at least one provider trained to perform cervical cancer screening (84%, 32/38) compared to clinics that did not report screening (28%, 9/32; PR 3.77, 95%CI 1.82–7.83).

**Conclusion:**

Integration of cervical cancer screening into FP clinics offers great potential to reach large numbers of reproductive-aged women. Increasing training of healthcare providers and ensuring adequate commodity supplies in FP clinics offer concrete solutions to increase screening in a largely unscreened population.

## Introduction

Cervical cancer is the most common cancer in sub-Saharan Africa (SSA). High human immunodeficiency virus (HIV) prevalence in Africa increases the risk of cervical cancer in a substantial proportion of women [1, 2]. In Kenya, cervical cancer represents 12% of the total cancer burden, but it is the leading cause of cancer death [3]. High HIV prevalence (6.6%) among Kenyan women contributes to the high incidence of cervical cancer [4–6]. Importantly, cervical cancer rates do not significantly decline despite antiretroviral treatment (ART) and immune reconstitution [7, 8]. The aging population of women living with HIV will continue to face a large lifetime risk of cervical cancer [9]. Fortunately, cervical cancer is preventable with appropriate screening and treatment of pre-cancers [10–13]. The Kenyan Ministry of Health (MOH) stresses the need to strengthen capacity, streamline, and standardize screening, detection, diagnosis, and treatment of cancers [14]. However, in the most recent Kenya Demographic and Health Survey (KDHS) in 2014, only 14% of women in Kenya had ever been screened for cervical cancer [15]. Potential barriers to cervical cancer screening include challenges with infrastructure, competing health priorities, lack of education about need for screening, low health literacy, and poverty [11, 16–20].

Kenyan national cancer screening guidelines recommend that any woman of reproductive age who presents to a healthcare facility for routine care should be screened using resource-appropriate methods [21]. At a national level, the Kenyan MOH has tasked family planning (FP) clinics (alongside HIV clinics) with the implementation of cervical cancer screening as a component of the overall cancer screening strategy. Specific data entry fields have been added to document screening in the hard-copy FP registers (MOH 512) that are used to document FP clinic visits [22]. In Kenya, FP clinics can be free-standing clinics or may be co-located in a larger facility with other clinic types such as antenatal care and HIV care. In Mombasa County, FP commodities are provided at no cost to FP clinics by the Department of Health (DOH), regardless of whether they are public or private facilities. Public FP clinics provide FP commodities at no cost to clients, while private clinics often charge a convenience fee. According to national guidelines, health facilities including FP clinics can and should perform cervical cancer screening if they have the necessary resources and training of the staff [21]. Facilities from level 2 (dispensaries and clinics) to level 5 (county referral hospitals and large private hospitals) can perform visual inspection with acetic acid (VIA) to screen for cervical cancer if they have trained personnel [23]. Papanicolaou smear is only recommended at level 4 (sub-county hospitals and medium-sized private hospitals) or level 5 facilities. Human papillomavirus (HPV) testing requires an accredited lab, which is generally only available at level 5 facilities and large private laboratories.

During the last Kenya Demographic and Health Survey in 2014, 58% of married women aged 15–49 and 65% of sexually active unmarried women used any method of contraception [15]. In Mombasa County, 44% of married women aged 15–49 years used a modern method of contraception. This illustrates that FP clinics are potentially a good venue to reach a substantial proportion of women, but it remains unclear how much cervical cancer screening is taking place in this setting. A survey of FP clinics in Mombasa County was conducted to understand current cervical cancer screening practices and explore clinic-level barriers and facilitators to screening.

## Methods

### Data collection

A cross-sectional study was conducted in a random sample of 70 (41%) FP clinics out of 170 FP clinics operating in Mombasa County in 2018. Study staff conducted structured interviews with FP clinic managers to collect information about each clinic, including size, location (urban, not urban), type (public, private with MOH-supported FP products, other), and presence of academic or non-governmental organization (NGO) support. Managers were also asked about clinic management practices, infrastructure, and cervical cancer screening practices. In clinics that provided cervical cancer screening, managers were asked about the availability of cervical cancer screening commodities (vaginal speculums, acetic acid, cotton swabs, and a light source). To understand staff characteristics, clinic managers were asked about their own experience and education level, the number and types of staff at the clinic, and the number of providers trained to perform cervical cancer screening. Study staff then asked clinic managers to list their top three challenges with cervical cancer screening.

Data were abstracted from the MOH 512 register for the 15-month period from October 1, 2017 until December 31, 2018. This register is provided only to FP clinics for documentation of FP clients visits according to standard procedures laid out by the Kenyan Ministry of Health and the register should be located within the FP clinic setting. Digital photographs were taken of the corresponding register pages with names masked. Study staff used these images to abstract data on number of clients who had cervical cancer screening conducted, the method used, the results of screening, and referral processes. Approved screening methods that were documented included VIA, visual inspection with Lugol’s iodine [VILI], HPV testing with VILI, HPV testing (alone), and Papanicolaou smear. From prior experience working with the MOH 512 register, a substantial amount of missing data was expected. In study planning discussions with members of the Mombasa County Health Management Team and County DOHS stakeholders, there was consensus that missing information on cervical cancer screening in the registers generally indicated that these services were not performed, and analyses were conducted with this assumption. However, some clinics used a separate register for cervical cancer screening. In clinics using an alternative register, these data were also captured. Data from the photographs of FP registers or other log books were transcribed to paper case report forms, then entered manually into a REDCap database [24]. All data collection forms were examined for missing responses and addended as needed. Following data entry, study staff performed a question-by-question assessment comparing hard copy data collection forms to the digital database to identify and correct any key-in errors.

### Data analysis

Characteristics of clinics, staff, and commodities were summarized using descriptive statistics as proportions or medians with interquartile ranges (IQR). Generalized linear models with a log link and binomial family were used to calculate prevalence ratios (PR) and identify clinic-level correlates of reporting any cervical cancer screening. Many of the potential correlates of cervical cancer screening were mechanistically related to one another. For example, number of clinicians and providers trained in cervical cancer screening are potential steps in the causal pathway linking facility type (public versus private) to cervical cancer screening outcome. For this reason, only unadjusted analyses are presented. An additional analysis was performed to identify clinic-level correlates of cervical cancer screening in the subset of clinics that documented any cervical cancer screening during the 15-month data collection period. All analyses were conducted using Stata version 17 (College Station, Texas) [25].

## Results

**Fig. 1:**
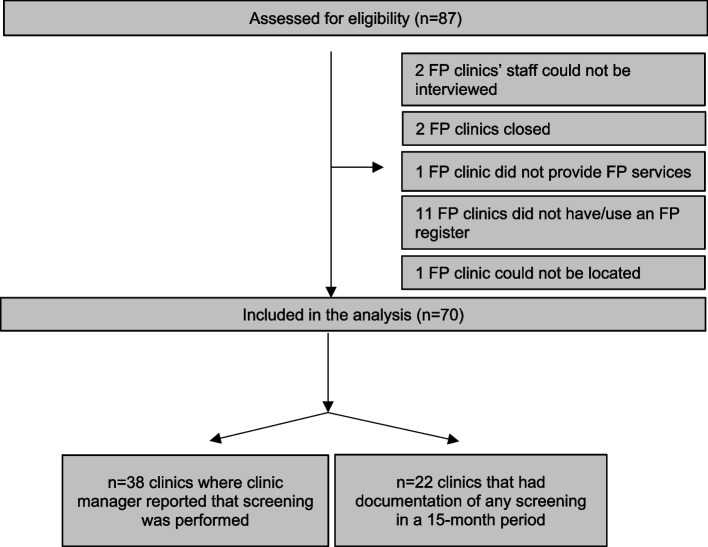
Family planning clinics in Mombasa County, Kenya assessed for eligibility between 1st October 2017 to December 31, 2018

A total of 70 clinics were included in this study (Table 1). An additional 17 FP clinics were assessed but were not included in this survey for several reasons (Fig. 1). These included clinic closure (*n* = 2), clinic did not provide FP services (*n* = 1), clinic did not have or did not use an FP register (*n* = 11), clinic could not be located (*n* = 1), or clinic staff were unable to be interviewed (*n* = 2). Of the 70 clinics, 54% (38) were urban and 63% (44) were private facilities with MOH-supplied FP commodities. Thirty (43%) of clinic had non-governmental organization support and 8 (11%) had academic institution support. The median number of staff was four (IQR 2–6) with a median of one (IQR 0–3) provider trained to perform cervical cancer screening. Clinic managers reported the top three challenge with providing cervical cancer screening were lack of supplies (27%, *n* = 19), lack of training (24%, *n* = 17), and clients declining cervical cancer screening (20%, *n* = 14). Most clinics held regular management meetings (87%, *n* = 61). Seventy-seven percent (54) of clinics had complete visual privacy and 70% (55) had complete auditory privacy to perform cervical cancer screening.

**Table 1:** Characteristics of all family planning clinics, clinics reporting and documenting cervical cancer screening in Mombasa County, 1st October 2017 to December 31, 2018

	All clinics (*n* = 70)	Any screening reported (*n* = 38)	Any screening captured over 15 months (*n* = 22)
Location:			
Urban	38 (54%)	21 (55%)	11 (50%)
Not urban	32 (46%)	17 (45%)	11 (50%)
Clinic type:			
Public	19 (27%)	14 (37%)	11 (50%)
Private	44 (63%)	21 (55%)	11 (50%)
Other	7 (10%)	3 (8%)	0 (0%)
Presence of non-governmental organization support	30 (43%)	20 (53%)	11 (50%)
Presence of academic institution support	8 (11%)	4 (11%)	0 (0%)
Median number of clinicians	4 (2–6)	5 (3–9)	5 (4–14)
Cervical cancer screening provided at no cost to client		19 (50%)	12 (55%)
How many providers are trained in cervical cancer screening?	1 (0–3)	2 (1–4)	2 (1–4)
Management meetings held	61 (87%)	35 (92%)	20 (91%)
Visual privacy?			
No privacy at all	3 (4%)	2 (5%)	0 (0%)
Partial/some privacy	13 (19%)	7 (18%)	5 (23%)
Complete privacy	54 (77%)	29 (76%)	17 (77%)
Auditory privacy?			
No privacy at all	3 (4%)	2 (5%)	0 (0%)
Partial/some privacy	12 (17%)	7 (18%)	4 (18%)
Complete privacy	55 (79%)	29 (76%)	18 (82%)

Of those clinics that reported any cervical cancer screening, most had dependable access to cotton swabs (92%, 35) and a light source (97%, 37) (Table 2). Dependable access to speculums (87%, 33) and acetic acid (71%, 27) were somewhat lower.

**Table 2:** Access to visual inspection with acetic acid (VIA) commodities in family planning clinics that reported they performed cervical cancer screening in Mombasa County, Kenya, 1st October 2017 to December 31, 2018

Variable	N (%) (*N* = 38)
Dependable access to speculums	33 (87%)
Dependable access to acetic acid	27 (71%)
Dependable access to cotton swabs	35 (92%)
Dependable access to light source	37 (97%)

Among the 70 clinics, 54% (*n* = 38) of clinic managers reported that their clinics performed cervical cancer screening. Prevalence ratios for factors associated with cervical cancer screening reported by the clinic manager are presented in Table 3. Public facilities represented 37% (14/38) of those reporting cervical cancer screening compared to only 16% (6/32) of clinics that did not report cervical cancer screening (PR 1.57, 95% confidence interval [CI] 1.05–2.33). There was no difference in the proportion of urban clinics among those that reported versus did not report cervical cancer screening (55%, 21/38 versus 53%, 17/32; PR 0.96, 95% CI 0.62–1.48). Non-governmental organization support was more common in clinics that reported screening (53%, 20/38) than in clinics that did not report any screening (31% 10/32; PR 1.48, 95% CI 0.97–2.27), though this difference was not statistically significant. Academic institution support did not differ between clinics that reported screening (11%, 4/38) versus those that did not report screening (13%, 4/32; PR 0.91, 95% CI 0.44–1.89). Four or more clinicians were on staff in 53% (20/38) of clinics that reported any cervical cancer screening, whereas only 22% (7/32) of clinics that did not report cervical cancer screening had four or more clinicians (PR 1.77, 95% CI 1.17–2.69). Clinics that reported cervical cancer screening were much more likely to have at least one provider trained to perform cervical cancer screening (84%, 32/38) compared to clinics not reporting cervical cancer screening (28%, 9/32; PR 3.77, 95% CI 1.82–7.83). The proportion of clinics holding management meetings was similar in clinics that reported (92%, 35/38) or did not report cervical cancer screening (81%, 26/38; PR 1.72, 95% CI 0.67–4.45).

**Table 3:** Correlates of cervical cancer screening reported by family planning clinic managers in Mombasa County, Kenya between October 1st 2017 to December 31, 2018

Variable	Any cervical cancer screening (*N* = 38)	No cervical cancer screening reported (*N* = 32)	PR (95% CI)	*p*-value
Public (as compared to non-public)	14 (37%)	5 (16%)	1.57 (1.05–2.33)	0.03
Urban (as compared to non-urban)	21 (55%)	17 (53%)	0.96 (0.62–1.48)	0.9
Presence of non-governmental organization support	20 (53%)	10 (31%)	**1.48 (0.97–2.27)**	0.07
Presence of academic institution support	4 (11%)	4 (13%)	**0.91 (0.44–1.89)**	0.8
More clinicians (dichotomized at median of 4)	20 (53%)	7 (22%)	1.77 (1.17–2.69)	0.007
At least one provider trained to perform cervical cancer screening	32 (84%)	9 (28%)	3.77 (1.82–7.83)	< 0.001
Management meetings held	35 (92%)	26 (81%)	1.72 (0.67–4.45)	0.3

*Abbreviations*: *PR* Prevalence ratio, *CI* Confidence interval

Thirty-one percent (*n* = 22) of clinics documented cervical cancer screening during the 15-month period. Public clinics represented 50% (11/22) of those FP clinics that documented any cervical cancer screening compared to 17% (8/48) of clinics that did not document cervical cancer screening (PR 2.68, 95% CI 1.49–5.14; Table 4). There was minimal difference in the proportion of urban clinics among those that reported versus did not report cervical cancer screening (50%, 11/22 versus 56%, 27/48; PR 1.19, 95% CI 0.60–2.37). The presence of NGO support did not differ significantly between clinics that documented cervical cancer screening (50%, 11/22) and those clinics without documented screening (40%, 19/48; PR 1.33, 95% CI 0.67–2.65). There were no clinics with academic institution support that documented any screening, so this PR could not be calculated. Four or more clinicians were present at 50% (11/22) of clinics that documented cervical cancer screening compared to 33% (16/48) of clinics that did not document screening (PR 1.59, 95% CI 0.80–3.15). Clinics that reported cervical cancer screening were much more likely to have at least one provider trained to perform cervical cancer screening (85%, 18/22) compared to clinics not reporting cervical cancer screening (48%, 23/48; PR 3.18, 95% CI 1.20–8.43). Finally, the proportion of clinics that held management meetings did not differ between clinics that documented screening (91%, 20/22) and clinics that did not document screening (85%, 41/48; PR 1.48, 95% CI 0.41–5.27).

**Table 4:** Correlates of cervical cancer screening documented in family planning registers or other log books in family planning clinics in Mombasa County, Kenya between October 1st 2017 to December 31, 2018

Variable	Any cervical cancer screening (*N* = 22)	No cervical cancer screening captured (*N* = 48)	PR (95% CI)	*p*-value
Public (as compared to non-public)	11 (50%)	8 (17%)	2.68 (1.40–5.14)	0.003
Urban (as compared to non-urban)	11 (50%)	27 (56%)	1.19 (0.60–2.37)	0.6
Presence of non-governmental organization support	11 (50%)	19 (40%)	1.33 (0.67–2.65)	0.4
Presence of academic institution support	0 (0%)	8 (17%)	--^a^	–
More clinicians (dichotomized at median of 4)	11 (50%)	16 (33%)	1.59 (0.80–3.15)	0.2
At least one provider trained to perform cervical cancer screening	18 (82%)	23 (48%)	3.18 (1.20–8.43)	0.02
Management meetings held	20 (91%)	41 (85%)	1.48 (0.41–5.27)	0.6

*Abbreviations*: *PR* Prevalence ratio, *CI* Confidence interval

^a^No clinics that recorded cervical cancer screening had any academic support

## Discussion

In Mombasa County, half of FP clinic managers reported that their clinics performed cervical cancer screening, but only a third of clinics documented any screening during the 15-month study period. Being a public facility and having at least one provider trained in cervical cancer screening were factors associated with both reported and documented cervical cancer screening.

Few studies have reported on integration of cervical cancer screening into FP services in Sub-Saharan Africa. A 2012 study of integration of cervical cancer screening into FP clinics in Mozambique identified several challenges including staff shortages, equipment problems, poor paper record systems, and difficulty following-up referred patients [26]. Similarly, a pilot study of the feasibility of cervical cancer screening in FP clinics in Eldoret, Kenya highlighted both reporting and documentation requirements and staffing as potential challenges [27]. The present study from Mombasa adds to this literature, emphasizing the importance of having sufficient numbers of trained staff to support integration of cervical cancer screening into FP clinics. In addition, a substantial proportion of FP clinics in Mombasa that reported that they provided cervical cancer screening also reported challenges with access to essential supplies including speculums and acetic acid.

In this survey, among those clinics that reported that they could perform cervical cancer screening, only 58% (22/38) documented any screening in a 15-month period. In the clinics that reported but did not document cervical cancer screening, it is possible that screening was being performed and not documented in a register that could be identified by research staff. However, this seems unlikely, as the research team asked about and collected data from sources other than the government-provided FP register if these were in use. An alternative possibility is that the managers in these clinics felt that they were prepared to offer cervical cancer screening, but no screening was actually taking place because of barriers such as lack of time, inadequate staffing, shortages of commodities, and clients declining screening [11, 16–20].

This study had important strengths. The survey included a large sample of both public and private FP clinics that captured screening practices, barriers, and facilitators that may be generalizable to other FP clinics in the region that are considering similar service integration. Combining interviews with register abstraction allowed us to identify both clinics that report offering screening and clinics that documented any cervical cancer screening during the 15-month observation period. This information allowed us to identify a potential gap between reported practices and actual performance.

There are a number of limitations to note. First, individual clients were not consistently identified with the same client identification numbers across multiple visits. As a result, it was not possible to track individual women to determine the proportion of all FP clients who received cervical cancer screening during the observation period or to evaluate the cervical cancer screening care cascade over time. Second, this study was not designed to assess FP client-level barriers to cervical cancer screening. Client-level factors like embarrassment, fear of screening, and lack of spousal support represent a distinct set of challenges that would not be overcome simply by addressing healthcare system barriers [28]. In addition, there could have been other reasons that clinics did not perform screening that were not assessed in this study. Third, this survey did not independently verify cervical cancer procedures but relied on self-report by clinic managers and documentation in the FP register. For example, clinic managers may have incorrectly reported cervical cancer screening due to social desirability bias, recall bias, and telescoping bias with temporal displacement of events. Incomplete documentation in program records is also a well-described limitation in implementation research [29, 30]. In this context, having both clinic manager report and clinic documentation allowed for triangulation, and may provide greater insight into actual practices than either measure alone. Fourth, we assess cervical cancer screening in FP clinics. Cervical cancer screening could have been conducted at other service delivery points that were not captured in this study. However, the goal was specifically to capture screening in FP clinics, as this is a delivery point that has great promise for reaching a large proportion of reproductive age women.

In conclusion, integration of cervical cancer screening into family planning clinics offers great potential to reach large numbers of reproductive-aged women. Current implementation of cervical cancer screening has not yet met the goals of the Kenyan MOH or the Mombasa County DOHS where all FP clinics should be providing some level of screening. Addressing modifiable factors including training healthcare providers, ensuring adequate staffing, and providing dependable access to screening commodities, offers hope for increased cervical cancer screening in a largely unscreened FP clinic population.

## Data Availability

The datasets generated and/or analyzed during the current study are not publicly available due to restrictions from the Kenyatta National Hospital - University of Nairobi Ethics and Research Committee (KNH-UON ERC) but are available from the corresponding author on reasonable request. This study was conducted with approval from the Kenyatta National Hospital - University of Nairobi Ethics and Research Committee (KNH-UON ERC), which requires that we release data from Kenyan studies (including de-identified data) only after they have provided their written approval for additional analyses. As such, data for this study will be available from the corresponding author (Dr. McKenna C Eastment, mceast@uw.edu) upon request, with written approval for the proposed analysis from the KNH/UON ERC. Their application forms and guidelines can be accessed at https://erc.uonbi.ac.ke/. Please contact the KNH-UON ERC at principal-cae@uonbi.ac.ke.
